# The complete chloroplast genome of *Passiflora caerulea*, a tropical fruit with a distinctive aroma

**DOI:** 10.1080/23802359.2021.1872442

**Published:** 2021-02-11

**Authors:** Ying-Feng Niu, Shu-Bang Ni, Shi-Hong Liu, Jin Liu

**Affiliations:** Yunnan Institute of Tropical Crops, Xishuangbanna, China

**Keywords:** *Passiflora caerulea*, chloroplast genome, phylogenetic analysis

## Abstract

*Passiflora caerulea* is native to brazil. In recent years, the edible, medicinal, and ornamental value of *P. caerulea* has stimulated its wide cultivation in Southeast Asian countries, especially China. Because the chloroplast genome is rich with information about the species evolution as well as its genetic relationship to other species, the *P. caerulea* chloroplast genome was sequenced, assembled, and annotated in this study. The *P. caerulea* chloroplast genome is 151,362 bp in total with an overall GC content of 37.03%. It has a quadripartite structure, includes a large single-copy region (LSC, 85,623 bp), a small single-copy region (SSC, 13,397 bp), and two inverted repeat regions (IRa and IRb, 26,180 bp combined). There are 131 genes in the *P. caerulea* chloroplast genome, including 79 protein-coding genes, 37 tRNA genes, 8 rRNA genes, and 7 pseudogenes. Phylogenetic analysis of 29 *Passiflora* spp. showed that *P. caerulea* is most closely related to *P. edulis.* These results provide a considerable foundation for *P. caerulea* conservation genetics research.

*Passiflora caerulea* L. belongs to the family Passifloraceae L. and is popularly known as wild passion, blue passion flower, or red-pulp passion (Bandeira et al. 2016). Because it is shaped like an egg and has fruit juice the color of an egg yolk, it is also known as egg fruit. The species is native to Brazil (Dhawan et al. 2004) and valuable in the pharmaceutical industry for its aerial parts, including its leaves, flowers, fruits, and roots (Rathod et al. 2014). *Passiflora caerulea* contains the flavonoid chrysin, which is a sedative with action similar to diazepam, but its activity is ten times lower with no myorelaxant effect (Medina et al. 1990; Speroni et al. 1996). Moreover, animals that received *P. caerulea* infusion showed decreases in biomarkers associated with physiological stress, demonstrating the phototherapeutic action of this plant species (Karina et al. 2013). Accordingly, its fruit has been used as a diuretic, painkiller, and sedative, and its roots have also been used for their anthelmintic action (Dhawan et al. 2004).

In particular, *P. caerulea*’s juice has a special flavor, with a total of 68 aroma components identified in it (Pan et al. 2019). Specifically, *P*. *caerulea* has aromas typically associated with banana, pineapple, litchi, guava, mango, cranberry, strawberry, star fruit, and dozens of other fruits (Zhou et al. 2009), so it has also been referred to as the “king of fruit juice.” In addition, the pulp of *P. caerulea* is rich in protein, fat, reducing sugar, vitamins and essential amino acids, and its seed is rich in high-quality edible oil (Quiroga et al. 2000). Because of its combined edible, medicinal, and ornamental value, *P. caerulea* has recently become widely cultivated in China, and Southeast Asia more broadly.

Chloroplast genomes are rich with genetic information, and their sequences are thus often used as foundational data in research on the evolution of species and the phylogenetic relationships among species. It is characterized not only by its matrilineal inheritance, but also by its high conservation of gene sequences. The chloroplast genomes of hundreds of tropical crops have been sequenced in recent years, but the chloroplast genome of *P. caerulea* has not been reported until now. In this study, the chloroplast genome of *P. caerulea* was sequenced, assembled, and annotated.

The sequenced *P. caerulea* specimen was collected from the Xishuangbanna Tropical Flowers and Plants Garden (100.786661 E, 22.014452 N) and deposited in the herbarium of the Yunnan Institute of Tropical Crops (Xishuangbanna, China) under the specimen voucher number YITC-2020-FZ-P-023. Its genomic DNA was extracted using the DNeasy Plant Mini Kit (Qiagen), and the quality and quantity of the genomic DNA were characterized using gel electrophoresis and the Nano-Drop 2000 spectrometer (Thermo Fisher Scientific, Waltham, MA, USA), respectively. DNA library construction followed the manufacturer's instructions (Illumina, San Diego, CA, USA) with insert sizes of 350 bp, and paired-end (PE) sequencing was conducted using the Illumina HiSeq 2500 platform). The clean reads were assembled with SPAdes-3.5.0 (http://soap.genomics.org.cn/soapdenovo.html) based on sequence overlap and paired-end relationships, the Kmer values used were Kmer 79 and Kmer 97 . To validate the assembly, the 4 boundaries of the IR region have been verified by Sanger sequencing, and the Sanger sequencing results are consistent with the second-generation sequencing assembly results. Annotation was performed using CpGAVAS2 (Shi et al. 2019) and ORF Finder (https://www.ncbi.nlm.nih.gov/orffinder/). To validate the correctness of gene annotation, putative gene and protein sequences were BLAST searched in the Nt (nucleotide databases) and Nr (non-redundant protein sequences) NCBI databases (https://www.ncbi.nlm.nih.gov/). The tRNA genes were further verified using the online tools ARWEN (Version 1.2, http://mbio-serv2.mbioekol.lu.se/ARWEN/) (Laslett and Canbäck 2008) and tRNAscan-SE 2.0 (http://lowelab.ucsc.edu/tRNAscan-SE/) (Lowe and Chan 2016). The chloroplast genome data of *P. caerulea* was uploaded to GenBank (http://www.ncbi.nlm.nih.gov/) under the accession number MT884000.

The double-stranded and circular complete chloroplast genome of *P. caerulea* was 151,362 bp in total, including 46,942 A bases (31.01%), 48,371 T bases (31.96%), 27,391 G bases (18.10%), and 28,658 C bases (18.93%). Like most other plants (Li et al. 2020; Niu et al. 2020), the chloroplast genome of *P. caerulea* has a quadripartite structure, includes a large 85,623-bp single-copy region (LSC) and a small 13,397-bp single-copy region (SSC) separated by two inverted repeat regions (IRa and IRb), each 26,180 bp in length. Unlike *Passiflora capsularis* and *Passiflora costaricensis*, the IR region of the chloroplast genome of *P. caerulea* is not lost (Cauz-Santos et al. 2020). The overall GC content of the whole *P. caerulea* chloroplast genome is 37.03%, with corresponding values of the LSC, SSC, and IR regions of 34.79%, 31.55%, and 42.10%, respectively. Additionally, the *P. caerulea* chloroplast genome was determined to encode a total of 131 genes, including 79 protein-coding genes, 37 tRNA genes, 8 rRNA genes, and 7 pseudogenes.

To examine the phylogenetic relationship of *P. caerulea* to its congeners, a maximum likelihood tree based on the complete chloroplast genome sequences of *P. caerulea* and 28 other *Passiflora* spp. was constructed with *Adenia mannii* (family Passifloraceae L.) used as the out group (Figure 1). The chloroplast genome sequences of 29 species were downloaded from GenBank (accession numbers shown in Figure 1). Multiple sequence alignment was conducted with MAFFT (Katoh and Standley 2013), and the phylogenetic analysis was conducted with RAxML8.2.4 (Stamatakis 2014). Node support was estimated from the results of 1000 bootstrap replicates. In the phylogenetic tree, most nodes had high bootstrap support values exceeding 84. The phylogenetic analysis indicated that *Passiflora edulis* is most closely related to *P. caerulea.* This study provides abundant genomic data for the conservation genetics of *P. caerulea* as well as for future phylogenetic studies of the genus *Passiflora* and the family Passifloraceae more broadly.

**Figure 1.**
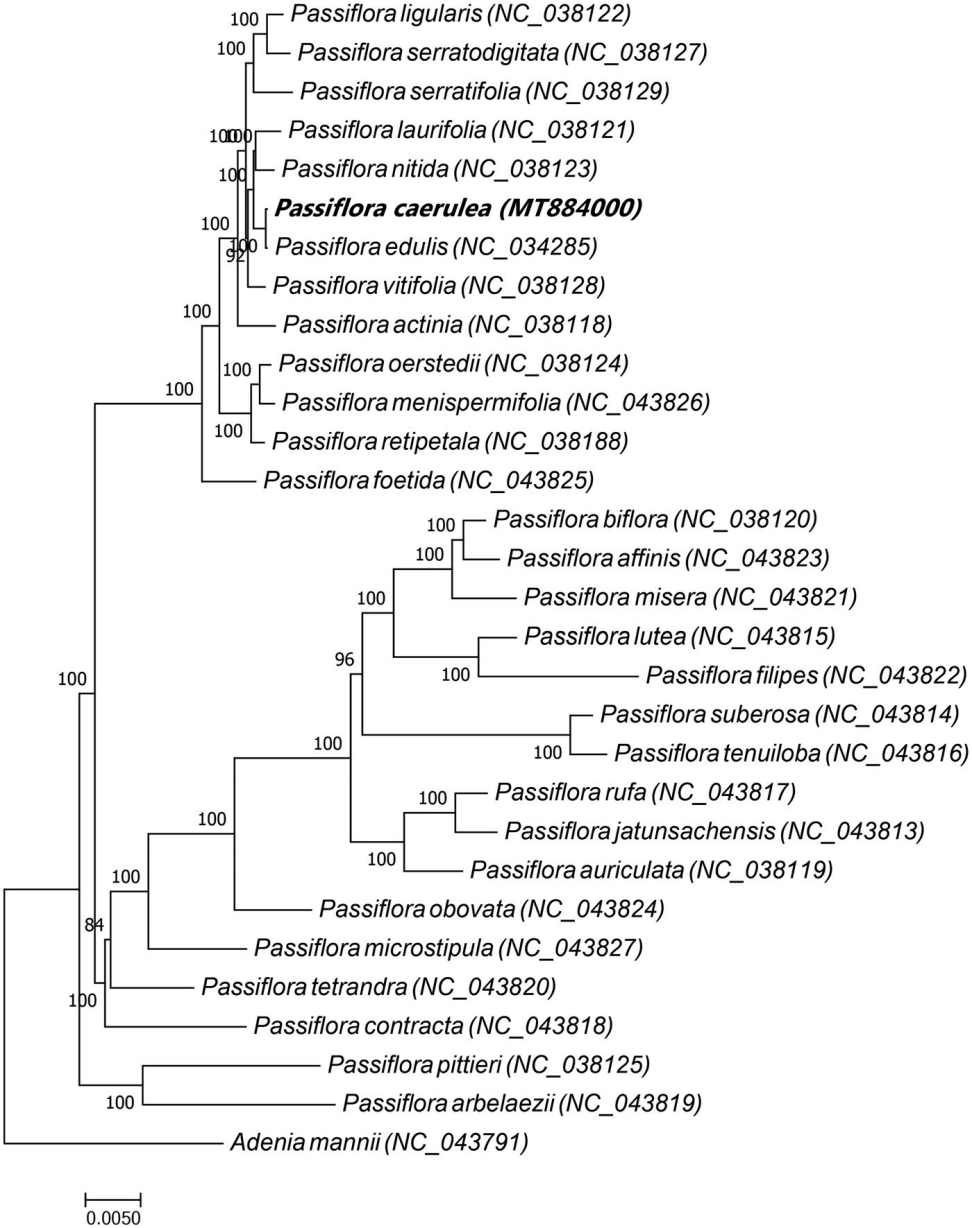
Maximum likelihood tree based on the complete chloroplast genome sequences of *P. caerulea* and 28 other *Passiflora* spp. with *Adenia mannii* (family Passifloraceae) was used as the out group. The species and GenBank accession numbers of the 30 chloroplast genomes for the ML tree construction has also been mentioned in Figure 1. *Passiflora ligularis* (NC_038122), *Passiflora serratodigitata* (NC_038127), *Passiflora serratifolia* (NC_038129), *Passiflora laurifolia* (NC_038121), *Passiflora nitida* (NC_038123), *Passiflora caerulea* (MT884000), *Passiflora edulis* (NC_034285), *Passiflora vitifolia* (NC_038128), *Passiflora actinia* (NC_038118), *Passiflora oerstedii* (NC_038124), *Passiflora menispermifolia* (NC_043826), *Passiflora retipetala* (NC_038188), *Passiflora foetida* (NC_043825), *Passiflora biflora* (NC_038120), *Passiflora affinis* (NC_043823), *Passiflora misera* (NC_043821), *Passiflora lutea* (NC_043815), *Passiflora filipes* (NC_043822), *Passiflora suberosa* (NC_043814), *Passiflora tenuiloba* (NC_043816), *Passiflora rufa* (NC_043817), *Passiflora jatunsachensis* (NC_043813), *Passiflora auriculata* (NC_038119), *Passiflora obovata* (NC_043824), *Passiflora microstipula* (NC_043827), *Passiflora tetrandra* (NC_043820), *Passiflora contracta* (NC_043818), *Passiflora pittieri* (NC_038125), *Passiflora arbelaezii* (NC_043819), *Adenia mannii* (NC_043791).

## Data Availability

The chloroplast genome sequence data that support the findings of this study are openly available in GenBank at https://www.ncbi.nlm.nih.gov/, reference number MT884000. The raw sequencing data are openly available in SRA database with the accession number PRJNA669899 and SRR12904124.
